# Combination therapy of chemokine receptor inhibition plus PDL-1 blockade potentiates anti-tumor effects in a murine model of breast cancer

**DOI:** 10.1186/2051-1426-3-S2-P227

**Published:** 2015-11-04

**Authors:** Heiyoun Jung, Ashley Bischof, Karen Ebsworth, Linda Ertl, Thomas Schall, Israel Charo

**Affiliations:** 1 Chemocentryx, Mountain view, CA, USA

## 

Trafficking and expansion of myeloid derived suppressor cells (MDSCs) plays a major role in immune suppression of tumor. MDSCs express chemokine receptors which likely mediate their recruitment to the tumor microenvironment. Suppression of T cells is also mediated by the interaction of programmed death-1 (PD-1) and its ligands which are abundantly expressed in cancer cells and immune infiltrates, including MDSCs. Here, we show that targeting both pathways through administration of a small molecule chemokine receptor antagonist (CCX9588 which blocks CCR1) and an anti-PDL1 antibody significantly reduced tumor burden in an orthotopic breast cancer mouse model. Primary tumor growth and lung metastasis were modestly reduced by either agent (anti-PDL-1 or CCR1 inhibitor) alone, but the combination of CCR1 inhibitor plus anti-PDL1 antibody resulted in significantly reduced tumor burden as compared to either of the single agents. Orthotropic breast cancer cell engraftment induces robust expansion of CD11b+Ly6Ghi Ly6Chi MDSCs, a subpopulation of which express CCR1. Analysis of the tumor infiltrating cells revealed that CCX9588 significantly reduced the number of MDSCs in primary tumors, suggesting that CCR1 blockade of MDSC trafficking translates into reduced tumor burden. Finally, analysis of human breast cancer patient samples from The Cancer Genome Atlas (TCGA) database revealed that the CCR1 ligands, CCL3 (MIP-1a) and CCL5 (RANTES) are present at significantly higher levels in breast cancers as compared to normal breast tissue. These data are the first to show that CCR1 chemokine receptor antagonists can act synergistically with PDL-1 inhibitors, and suggest a novel approach for potentiating the activity of immune cell checkpoint inhibitors.

